# Small bowel emergency surgery: literature's review

**DOI:** 10.1186/1749-7922-6-1

**Published:** 2011-01-07

**Authors:** Carlo Vallicelli, Federico Coccolini, Fausto Catena, Luca Ansaloni, Giulia Montori, Salomone Di Saverio, Antonio D Pinna

**Affiliations:** 1 General, Emergency and Transplant Surgery Dept., Sant'Orsola-Malpighi University Hospital, Bologna, Italy; 2 General and Emergency Surgery Dept., Ospedali Riuniti Hospital, Bergamo, Italy; 3 Emergency and Trauma Surgery Dept., Maggiore Hospital, Bologna, Italy

## Abstract

Emergency surgery of the small bowel represents a challenge for the surgeon, in the third millennium as well. There is a wide number of pathologies which involve the small bowel. The present review, by analyzing the recent and past literature, resumes the more commons. The aim of the present review is to provide the main indications to face the principal pathologies an emergency surgeon has to face with during his daily activity.

## Review

The small intestine is a complex organ with several functions. In fact it is capable of digestion, absorption and secretion, endocrine function and protects the internal environment against noxious ingested substances and against luminal bacteria and their toxins. The potential surface area available for digestion and absorption is amplified 600-times by circular mucosa folds, villus mucosal architecture and the microvillus surface of epithelium. Although specific properties are characteristic of specific segments of small bowel, like bile acid absorption in distal ileum, maximal resections are feasible without a significant morbidity because of the compensatory adaptation of remaining intestine. The small bowel measures about 120 cm in length from pylorus to ileocecal valve. The jejunum begins at ligament of Treitz. Jejunum and ileum are suspended by a mobile mesentery covered by a visceral peritoneal lining that extends onto the external surface of the bowel to form the serosa. Jejunum and ileum receive their blood from the superior mesenteric artery (SMA). Although mesenteric arcades form a rich collateral network, occlusion of a major branch of the SMA may result in segmental intestinal infarction. Venous drain is via the superior mesenteric vein, which then joins the splenic vein behind the neck of the pancreas to form the portal vein. Peyer's patches are lymphoid aggregates present on the antimesenteric border of distal ileum. Smaller follicles are present through all small bowel. Lymphatic drainage of intestine is abundant. Regional lymph nodes follow the vascular arcades and then drein toward the cysterna chyli. Jejunal and ileal wall consists of serosa, muscolaris, submucosa and, innermost, mucosa [1].

### Mechanical small bowel obstruction

Acute mechanical obstruction of the intestine is a common surgical emergency and a major cause of admission to emergency surgery departments. Small bowel obstruction occurs when there is an obstacle to the flow of luminal contents caused by an extrinsic or intrinsic encroachment on the lumen [2]. Adynamic ileus presents the same symptoms of mechanical obstruction but the underlying problem is disordered motility. One of the keys to management of intestinal obstruction is early diagnosis. Particularly, accurate early recognition of strangulation is crucial because this emergency causes bowel ischemia, necrosis and perforation. In neonates most common causes are atresia, midgut volvulus and meconium ileus, in infants groin hernia, intussusception and Meckel's diverticulum, whereas in young adults and adults adhesions and groin hernia [1]. In small bowel obstruction the normal mechanisms of intestinal absorption are compromised, so an excess of fluid loss occurs. Initially vomiting, bowel wall edema and transudation into the peritoneal cavity are present, whereas in the later stages venous pressure increases with consequent bleeding into the lumen and aggravation of hypovolemia [2]. Diagnosis is usually clinical. Main symptoms are abdominal pain, absence of flatus or stool, nausea or vomiting, dehydration, and abdominal distension if the obstruction is not in proximal jejunum [1]. Moreover the kind of pain suggests the level of the small bowel obstruction. Proximal obstruction tend to present with more frequent cramps whereas distal obstructions cause less severe cramps with longer duration between episodes. Laboratory tests show an elevated hematocrit because of intravascular volume loss. A significant leukocytosis could indicate a strangulation or perforation. Plain X-rays of the abdomen reveal dilatation of the small bowel and air-fluid levels [3]. CT scan, eventually with oral contrast, shows the dilatation of proximal bowel and the collapse of distal bowel [4,5]. Also ultrasounds may be useful [6,7]. The key of management of small bowel obstruction is the identification of intestinal strangulation, because mortality increases from 2 to 10 folds in such cases. Therefore an immediate surgical repair with an eventual bowel resection is mandatory. However, the clinical diagnosis of small bowel strangulation is extremely difficult and CT scan becomes very useful, usually on the basis of either bowel wall thickening, mesenteric edema, asymmetrical enhancement with contrast, pneumatosis, or portal venous gas. Mortality for small bowel obstruction has decreased during the past 50 to 60 years from 25% to 5% [8-20]. Initial therapy aims at correction of depletion of intravascular fluids and electrolyte abnormalities. The patient should be given nothing by mouth and nasogastric tube should be inserted in patients with emesis. In patients with adhesive small intestine obstruction, water-soluble contrast medium (Gastrografin^®^) with a follow-through study has not only a diagnostic but also a therapeutic role, because it is safe and reduces the operative rate and the time to resolution of obstruction, as well as the hospital stay [21-23]. Surgical intervention is instead mandatory for patients with a complete small bowel obstruction with signs or symptoms indicative of strangulation, perforation or those patients with simple obstruction that has not resolved within 24 to 48 hours of non operative treatment [23]. The surgical approach includes adhesiolysis and resection of non viable intestine. The extension of intestinal resection depends on the purple or black discoloration of ischemic or necrotic bowel. Viable intestine also has mesenteric arterial pulsation and normal motility. When ischemic damage is more limited, is sufficient adhesiolysis followed by a 10-15 minutes period of observation to allow for possible improvement in the gross appearance of the involved segment.

The role of laparoscopy in small bowel obstruction has still to be defined. Certainly, laparoscopy represents a diagnostic act and sometimes has a therapeutic role [24,25]. The major indication is small bowel obstruction due to unique band adhesion without signs of ischemia and necrosis. In laparoscopic procedures the first trocar has to be positioned using Hasson's technique for open laparoscopy to avoid accidental bowel perforations related to bowel distension and adhesions with the abdominal wall. After that, two 5 mm trocars must be introduced under vision to explore the peritoneal cavity and find the bowel segment obstructed by the band adhesion. If ischemic or necrotic bowel is present conversion to open surgery may be necessary. An atraumatic grasp can be used to isolate the band adhesion, which is coagulated by bipolar coagulator and then sectioned with scissors. So the obstructed bowel segment is liberated. The rate of laparotomic conversions ranges widely from 0% to 52%, depending on patient selection and surgical skills [24-29]. The principle reason is a difficult exposition and treatment of band adhesions due to a reduced operating field caused by small bowel dilatation, multiple band adhesions, and sometimes the presence of posterior band adhesion which are more difficult to treat laparoscopically. The predictive factors for successful laparoscopic adhesiolysis are a number of previous laparotomies lower than 3, a non-median previous laparotomy, appendectomy as previous surgical treatment causing adherences, a unique band adhesion, an early laparoscopic management (possibly within 24 hours), no signs of peritonitis and the experience of the surgeon [24-29]. Relative contraindication are 3 or more previous laparotomies and multiple adherences. Finally, absolute contraindications to laparoscopic adhesiolysis are an abdominal film showing a remarkable dilatation (more than 4 cm) of the small bowel, signs of peritonitis, severe cardiovascular or respiratory co-morbidities and haemostatic disease, and hemodynamic instability. Laparotomic conversion is often related to a higher morbidity rate, so when the predictive factors for a successful laparoscopy are not present a primary laparotomic access becomes necessary [25]. In any case, early conversion is recommended to reduce postoperative morbidity [25]. Many studies in literature suggest that laparoscopic adhesiolysis in small bowel obstruction is convenient if performed by skilled surgeons in correctly selected patients, resulting in a shorter hospital stay with a early flatus and a early realimentation and in a lower postoperative morbidity. Nonetheless laparoscopic surgery requires a longer operating time and recurrent obstruction remains the major postoperative risk in the management of these patients.

### Crohn's disease

Acute surgical emergencies in patients with inflammatory bowel disease are infrequent but may be dangerous for life. Crohn's disease is an important cause of small bowel acute surgery [1,30-32]. Ileal localization, particularly terminal ileum, is the most frequent in Crohn's disease, despite its pan-intestinal nature. Skip lesions interest full-thickness the bowel wall and are able to induce a wide spectrum of acute surgical emergencies. Small bowel is the main site of bleeding in Crohn's disease. The bleeding is often from a localized source, caused by erosion of a blood vessel within multiple deep ulcerations that extend into bowel wall. Severe hemorrhage is rare and requires surgery [33,31]. Other surgical indications include a bleeding who doesn't slow after 4 to 6 units of blood and recurrent hemorrhage [1]. Because of segmental disease, the best approach is to localize the source of bleeding preoperatively. The patient is stabilized and a nasogastric tube is inserted. Gastroscopy, angiography and the use of labeled red cells scan help localizing the hemorrhage. If the site of bleeding is identified in small bowel, resection and primary anastomosis is the gold standard surgical treatment. Perforation is another surgical emergency in patients with Crohn's disease [33]. It occurs in 1% to 3% of cases. The transmural nature of Crohn's disease creates inflammatory adhesions between bowel and local structures, so the perforation is often sealed. If perforation is suspected, the patient must be resuscitated and prepared to surgery. Jejunal and ileal perforations require resection and primary anastomosis if possible [1,33,31,32]. Otherwise resection with intestinal diversion is necessary. More than 25% of patients undergoing surgery for Crohn's disease will have either an intra-abdominal mass or abscess, and 40% of these have an associated fistula [31]. An intra-abdominal mass may be the consequence of distended loops of proximal bowel caused by distant strictures, thinning of diseased loops, phlegmon with associated fistulae, or an abscess cavity [34,35]. The cause of abdominal abscesses is the transmural ulceration of the diseased bowel, which creates secondary adhesions to adjacent structures resulting in intraperitoneal, retroperitoneal or rarely intramesenteric abscesses. Progresses in interventional radiological techniques have increased, facilitating an improvement in patient's general conditions before the eventual surgical repair. If general conditions are favorable, in selected cases of perforation of the jejunum or ileum without abscess and early intervention, primary reconstruction is possible. However, having to do with intestinal perforation and abscessed small bowel, resection with fecal diversion is the gold standard surgical strategy. Intestinal obstruction is the main complication requiring surgical intervention in Crohn's disease, affecting 35% to 54% of patients [33,36,37]. Because of transmural nature of disease process, obstruction can be the consequence of an acute and active inflammation superimposing on a stenotic portion of the bowel. Fibrosis and scarring with stricture formation, and mass effect of an adjacent abscess or phlegmon are common events in Crohn's disease. Although it is rare, a complete or near complete intestinal obstruction not responsive to medical therapy requires a surgical treatment [38,39]. The treatment may be a resection or a strictureplasty depending on localization of the disease [34,31]. Strictureplasty is a safe and efficacy procedure for small bowel Crohn's disease in the long term [33,40]. Strictureplasty should be reserved only for fibrotic stricture with inactive disease and only if resection is inappropriate [33,41]. Resection has been for a long time the mainstay treatment of Crohn's disease associated with small bowel strictures. However, recurrence rates are high and most of patients need multiple resections. So, the concern of short-bowel syndrome led to the use of bowel-sparing procedures. Principle indications for strictureplasty are multiple strictures over large length of bowel, previous resections, short bowel syndrome and strictures associated with phlegmon or fistula [34,31,42]. Contraindications include preoperative malnutrition (albumin < 2 g/dL), perforation, multiple strictures over short length of bowel, stricture short distant from area of resection and bleeding from planned strictureplasty site [34,31,42]. Several strictureplasty techniques have been described and the choice depends on the length of the stricture [34]. Short strictures are treated with Heineke-Mikulicz strictureplasty. A longitudinal enterotomy is realized over the stricture on the antimesenteric border of the bowel and extended 1 to 2 cm onto either side of normal bowel. The enterotomy can be realized using bistury or cautery. Then, the enterotomy is closed transversally with a interrupted, sieromuscolar, absorbable suture. The closure should be performed in one or two layers and must be tension-free. The Finney strictureplasty is used for strictures of intermediate length. First of all, a stay suture is localized in the midpoint of the stricture. The enterotomy is performed throught the stricture, again extending 1 to 2 cm onto normal bowel. Then strictured segment is folded onto itself to realize a "U" and another stay suture is localized in the normal side of bowel to keep the "U" in place. The posterior edges are sutured in a continuous way using an absorbable suture. In the end, the anterior edges are closed with a interrupted non absorbable suture. In 1996, Michelassi introduced the side-to-side isoperistaltic strictureplasty for long strictures, usually greater than 20 to 25 cm, and multiple strictures over a limited area [43]. In this technique, the sctrictured bowel is lifted up and his mesentery is divided at the midpoint. Then the diseased bowel is divided between atraumatic bowel clamps at the midpoint of the stricture. The proximal end of the cut bowel is brought over the distal end in a side-to-side way. The two loops are approached with a single-layer, interrupted, non absorbable suture. Then enterotomy is realized longitudinally for the length of the stricture. The ends of bowel are spatulated to avoid blind ends. Next, a inner layer of running, full-thickness, absorbable suture is placed and continued anteriorly. This anterior layer is then followed by a layer of interrupted, non absorbable, sieromuscolar suture. Markedly thickened bowel loops, thickened and friable mesentery, inflammatory phlegoms, fistula, abscesses and adhesions from previous surgery represent a surgical challenge to the laparoscopic approach. Many studies in literature suggest that laparoscopic approach is feasible and safe in terminal ileal Crohn's disease, because it offers advantages in terms of pulmonary function, length of hospital stay, duration of postoperative ileus, cosmesis, postoperative small bowel obstruction, and early postoperative complications. Furthermore laparoscopy reduces the hospitalization costs and improves patient satisfaction [44][32][45-47].

### Small bowel neoplasms

Tumors of the small bowel are a very rare entity, accounting for only 1% of all gastrointestinal neoplasms and 0,3% of all tumors [48-51]. The most common modes of presentation are intestinal obstruction and occult gastrointestinal hemorrhage. Occasionally, the presentation involves the development of a palpable but otherwise asymptomatic mass, whereas perforation and gross bleeding are rare. Small bowel tumors are usually located in the proximal small bowel, with the exception of adenocarcinoma in the contest of ileal Crohn's disease and NETs [1,52,50,51,54].

Adenomas are the most common benign tumors of jejunum and ileum. Their histological subtype are either tubular adenomas with low malignant potential or villous adenomas with high malignant potential. Lipomas are more frequent in the ileum, have no malignant potential and do not require a surgical excision unless symptomatic.

Malignant neoplasm present similarly to benign lesions. Diagnosis is often delayed conducing to advanced tumors, for whom surgical resection is rarely curative [1,55-57]. Adenocarcinomas represent 50% of all small bowel malignancies [1]. Most lesions are located in the proximal bowel, except in the setting of Crohn's disease in which most are ileal [1,57,58]. Resection is the best treatment but overall the prognosis is poor due to late presentation in most patients (15% to 35% 5-year survival) [1,58]. Lymphomas represent 10% to 20% of small bowel malignant tumors. The ileum is the most common site of involvement because of the greatest amount of gut-associated lymphoid tissue [1]. Primary small-bowel lymphoma is the most common extranodal form of lymphoma. Most are non-Hodgkin's lymphomas and predominantly B-cells in origin [59-62]. Patients commonly present with fatigue, weight loss and abdominal pain, whereas perforation, bleeding, obstruction or intussusceptions are less frequent. Treatment in such emergent cases is surgical and consists in resection along with a wedge of mesentery. Adjuvant therapy is recommended for patients with positive margins. Survival for completely resected intestinal lymphomas is about 50% [1].

Gastrointestinal stromal tumors (GISTs) can arise anywhere in the gastrointestinal tract: 50-70% in the stomach, 20-40% in the small bowel, 5-15% in the colon and rectum, 5% in the esophagus and the omentum, and rarely in the mesentery or retroperitoneum [52,63-67]. They account for approximately 0,1% to 3% of all gastrointestinal neoplasms. GISTs are more common between the ages of 40 and 70, without sex difference. GISTs are thought to arise from the intestinal cells of Cajal, which are intestinal pacemaker cells that regulate peristalsis. Bleeding occurs in almost 50% of GISTs. Approximately 35% of patients present with abdominal mass causing or not symptoms, and 20% of patients have abdominal pain. When tumors arise from the small bowel slow bleeding and mild obstructive symptoms can go undiagnosed for a long. GISTs usually do not metastatize beyond the gastrointestinal tract and the liver [68,69]. Prognosis varies and depends on the site of GIST, origin, mitotic index, and size. Small intestine GISTs are more aggressive and have a worst prognosis [70,71]. When GIST presents as an emergency, surgery is the mainstay. In cases where is feasible and the risk-benefit balance is favourable, the goal is to completely resect the primary tumor, surrounding normal tissue, and adjacent organs if they are affected with GIST. Because of their fragility, surgeon must handle GIST with great care to avoid tumor rupture. GISTs are resistant to chemotherapy and radiotherapy [52]. However targeted chemotherapy has dramatically increased the outcome of GISTs treatment, either of non-resectable GISTs.

Gastroenteropancreatic neuroendocrine tumors (GEP-NET) are a heterogeneous group of uncommon malignancies occurring in the gastrointestinal system. The incidence of GEP-NET is 2 to 3 per 100,000 people per year [72,73]. Symptoms depend on the tumor cells of origin and the effects of secreted substances. However, patients may seek medical care when gastrointestinal emergencies occur. Imaging studies help to make a diagnosis and include ultrasounds, CT, RMI, PET, and radiolabeled somatostatin receptor scintigraphy (OctreoScan) [72]. Small bowel NETs are the most common and occur more frequently in ileum than in jejunum. Unfortunately 60% of these neoplasms are diagnosed when distant metastasis to lymph nodes and liver have occurred. 5-years survival rate is 60%, but drops to 30% if liver metastasis are present [72,74]. About 10% of patients with metastatic ileal NETs have classic carcinoid syndrome. Occasionally, ileal NET presents with a massive gastrointestinal bleeding, secondary to sclerosis of vasa recta, due to hypersecretion of serotonin. Sclerosis of arterial vessels may also provoke a bowel ischemia. Otherwise, endo-luminal growth of the cancer or mesenteric fibrosis create the condition for an intestinal obstruction. In such cases surgical treatment becomes emergent.

Intestinal involvement of metastatic cancer is common, mostly in the form of peritoneal carcinomatosis. Because of the continuous recirculation of peritoneal fluid through all the abdomino-pelvic cavity, small bowel is an elective site for peritoneal metastasis. All abdominal tumors can lead to peritoneal carcinomatosis, particularly colorectal cancer, ovarian cancer, gastric cancer, and primitive peritoneal neoplasms. The diagnosis of peritoneal secondary tumors as the cause of small bowel obstruction is often difficult. Obstruction in these circumstances never resolves by conservative treatment and surgical intervention is almost always indicated. Although peritoneal carcinomatosis has been for a long considered a terminal condition, in the latest years a new curative option consisting of extensive cytoreductive surgery (CRS) and hyperthermic intraperitoneal chemotherapy (HIPEC) has emerged for accurately selected patients [75-78].

### Meckel's diverticulum and acquired jejunoileal diverticulosis

Meckel's diverticulum is the most common congenital malformation of the gastrointestinal tract, interesting 2% to 4% of population [79]. It is a true diverticulum due to the persistence of omphalo-mesenteric duct, which connects in fetal life the yolk sac to the intestinal tract and usually obliterates in the 5^th ^to 7^th ^week of life. It is localized on anti-mesenteric border of the distal ileum, usually 30-40 cm far from the ileo-cecal valve [1,79,80]. Meckel's diverticulum is lined mainly by the typical ileal mucosa as in the adjacent small bowel. However, in 20% of cases ectopic gastric mucosa may be found. Globally the incidence of complications ranges from 4% to 16% [79]. Although there is no sex differences in the incidence of Meckel's diverticulum, its complications are 3-4 times more frequent in males. Meckel's diverticulum is the most common cause of bleeding in the pediatric age group. The risk of complications decreases with increasing age [79,80]. The most frequent complications in adults are obstruction due to the intussusceptions or adhesive band, ulceration, diverticulitis and perforation [79,1,80]. Preoperative diagnosis of symptomatic Meckel's diverticulum is difficult, especially in patients with symptoms other than bleeding. In doubtful cases, laparoscopy is the preferred diagnostic modality. However, technetium 99-m pertechnate scan is the most common and accurate non-invasive investigation, although it is specific to ectopic gastric mucosa, not to Meckel's diverticulum [80]. In the presence of symptoms, the treatment of choice is the surgical resection. This can be achieved either by diverticulectomy or by the segmental bowel resection and anastomosis, especially when there is palpable ectopic tissue, intestinal ischemia or perforation [1,79].

Acquired jejunoileal diverticulosis (JID) is a rare entity often asymptomatic and treated conservatively. However, JID can develop a number of complications requiring acute surgical care [81-83]. The incidence of JID increases with age, with the peak occurring in the sixth and seventh decades of life. The etiology is unclear, but the most commonly accepted is the one related to the acquired mechanism. A motor dysfunction or jejuno-ileal dyskinesia leads to an intraluminal pressures increase. As a result, mucosa and submucosa herniate through the weakest site of the muscolaris of the small bowel, which is on the mesenteric border where paired vasa recta penetrate the bowel wall [81,84]. So, these are pseudodiverticula. About 55% to 80% of diverticula occur in the jejunum, 15% to 38% in the ileum and 5% to 7% in both [85,86]. Two-third of patients have multiple diverticula and therefore a major risk of developing complications [85]. Although the diagnosis of JID is often accidentally, 10% to 19% of patients with JID present with acute and emergent complications. Diverticulitis occurs in 2% to 6% of patients and can progress to gangrene with full-thickness necrosis and perforation, which has a mortality rate as high as 40%. Perforation presents either with localized or generalized peritonitis, and the mainstay of treatment includes resection of the affected segment and primary anastomosis. Obstruction occurs in 2% to 4% of patients, due to adhesions, intussusceptions, volvolus, extrinsic compression from a fluid-filled diverticulum, enteroliths [81,85]. Bleeding complications interest 3% to 8% of patients with JID. The proximity of the neck of the diverticula to the mesenteric vessel is responsible for bleeding resulting from erosion and ulceration of the mucosa. In case of massive hemorrhage, surgical resection of the affected bowel and anastomosis is mandatory [81,86].

### Acute mesenteric ischemia

Acute mesenteric ischemia (AMI) is an uncommon event, according for less than 1 case in every 1000 hospital admissions. Females are affected with three times the frequency of males and patients are usually between the age of 60 and 70 with several comorbidities [81]. Arterial embolism is the major cause of AMI, according for 40% to 50% of cases [87]. Most events are thromboembolic and arise from a cardiac source [87]. Thromboemboli tend to lodge in proximal superior mesenteric artery (SMA), just beyond the first jejunal branches, a minority (15%) may lodge at the SMA origin, whereas about 50% lodge distal to the middle colic artery [88,89]. In this case, proximal intestine and ascending colon are spared. Instead atheroembolic emboli tend to be smaller and to lodge in the distal SMA, therefore affecting bowel perfusion less often and in more localized areas. Acute arterial thrombosis superimposed on preexisting severe atherosclerotic disease accounts for 25% to 30% of all cases [87,90]. Bowel infarction is more insidious because extensive collateral are able to maintain viability until there is a final closure of critically stenotic vessel or collateral. The infarction is more confluent, without sparing of small bowel or right colon circulation, because SMA is often interested at its origin. Acute presentation on a history of cronic mesenteric ischemia is usual. The small bowel is able to tolerate a significant reduction in blood flow. However, when the ischemia is prolonged, it leads to disruption of the intestinal mucosa. Patients present abdominal pain. SMA embolism has the more rapid clinical decline due to the lack of collateral vessels. The advent of high-quality computed tomography angiography has supplanted angiography to make the diagnosis of AMI [91,92]. However angiography still plays an important role not only in the diagnosis but also in the treatment [93]. Diagnostic laparoscopy is not widely accepted because it may miss areas of nonviable bowel. After initial resuscitation and stabilization of the patient, surgery is required for all patients who have evidence of threatened bowel. Surgeon should proceed with revascularization before resecting any intestine unless faced with an area of frank necrosis or perforation or peritoneal soilage. In such cases resection of the affected bowel without reanastomosis and containment of the spillage should be rapidly achieved before revascularization. In few patients with massive bowel necrosis revascularization can be avoided.

### Miscellaneous conditions

Pneumatosis intestinalis is the presence of gas within the abdominal wall of the bowel. Benign pneumatosis is an incidental finding without any underlying pathology. Conversely, when pneumatosis intestinalis is the result of primary intestinal pathology, urgent surgery is mandatory. The intramural gas can result from necrosis caused by ischemia, infarction, neutropenic colitis, volvulus, and necrotizing enterocolitis. Benign pneumatosis instead is related to a pulmonary source in patients with COPD, asthma, or cystic fibrosis. The intrathoracic air can dissect via the retroperitoneum and into the intestinal wall. It is generally accepted that patients with pneumatosis intestinalis associated with either bowel obstruction or ischemia usually require urgent surgery [94]. The presence of air within the bowel wall itself does not mandate resection, because the air may have tracked from another site within the bowel, such a segment of ischemia or necrosis. In such a case, only the ischemic bowel segment must be resected [1].

Small bowel ulceration is usually the result of ingested medications like enteric-coated potassium chloride, non-steroidal anti-inflammatory drugs, and corticosteroids [1,95]. Clinical presentation is usually an intermittent small bowel obstruction. Preoperative localization of these lesions is difficult, and is frequently necessary the palpation of the small bowel at laparotomy or an intraoperative endoscopy. The treatment of small bowel ulceration is surgical resection. Suture repair after the perforation of small bowel ulceration presents a high rate of complications. Recurrence after resection is rare.

The accidental or intentional ingestion of foreign bodies is not rarely observed in emergency departments. Although intestinal perforation is rare, the development of abdominal pain with tenderness and leukocytosis strongly suggests a perforation. In case of perforation, surgical resection is required, because antibiotic treatment is associated with chronic infection or stricture formation.

## Competing interests

The authors declare that they have no competing interests.

## Authors' contributions

VC, CoFe: Contributed both as first author, participating in study conception, in analysis and interpretation of data, in manuscript draft and revision and in giving the final approval.

AL, CF, MG, SDS, PAD: Participate in manuscript draft and revision and in giving the final approval.
